# Supplementary material for: Winter wheat yield prediction using convolutional neural networks from environmental and phenological data
Source: Sci Rep. 2022 Feb 25;12:3215. doi: 10.1038/s41598-022-06249-w (PMC8881605; doi:10.1038/s41598-022-06249-w)
Supplement: Supplementary file 1 — Supplementary Information. [file 41598_2022_6249_MOESM1_ESM.pdf]

Supplementary material of the manuscript, Winter Wheat Yield Prediction Using Convolutional Neural Networks from Environmental and Phenological Data

Table S1. Sources of the data used in the study

Data types	Source	Links
Weather data	Deutscher Wetterdienst (DWD)	https://www.dwd.de/DE/klimaumwelt/klimaueberwachung /phaenologie/daten_deutschland/jahresmelder/ jahresmelder_node.html
Soil data	BÜK1000N (BGR)	https://www.bgr.bund.de/EN/Themen/Boden/Projekte/ Informationsgrundlagen_abgeschlossen/BUEK1000/ BUEK1000_en.html
Crop yield	Regionaldatenbank Deutschland, 2020	www.regionalstatistik.de/genesis/online
Crop phenology data	Phenology database (DWD)	https://www.dwd.de/DE/leistungen/phaeno_sta/ phaenosta.html?nn=16102#buehneTop

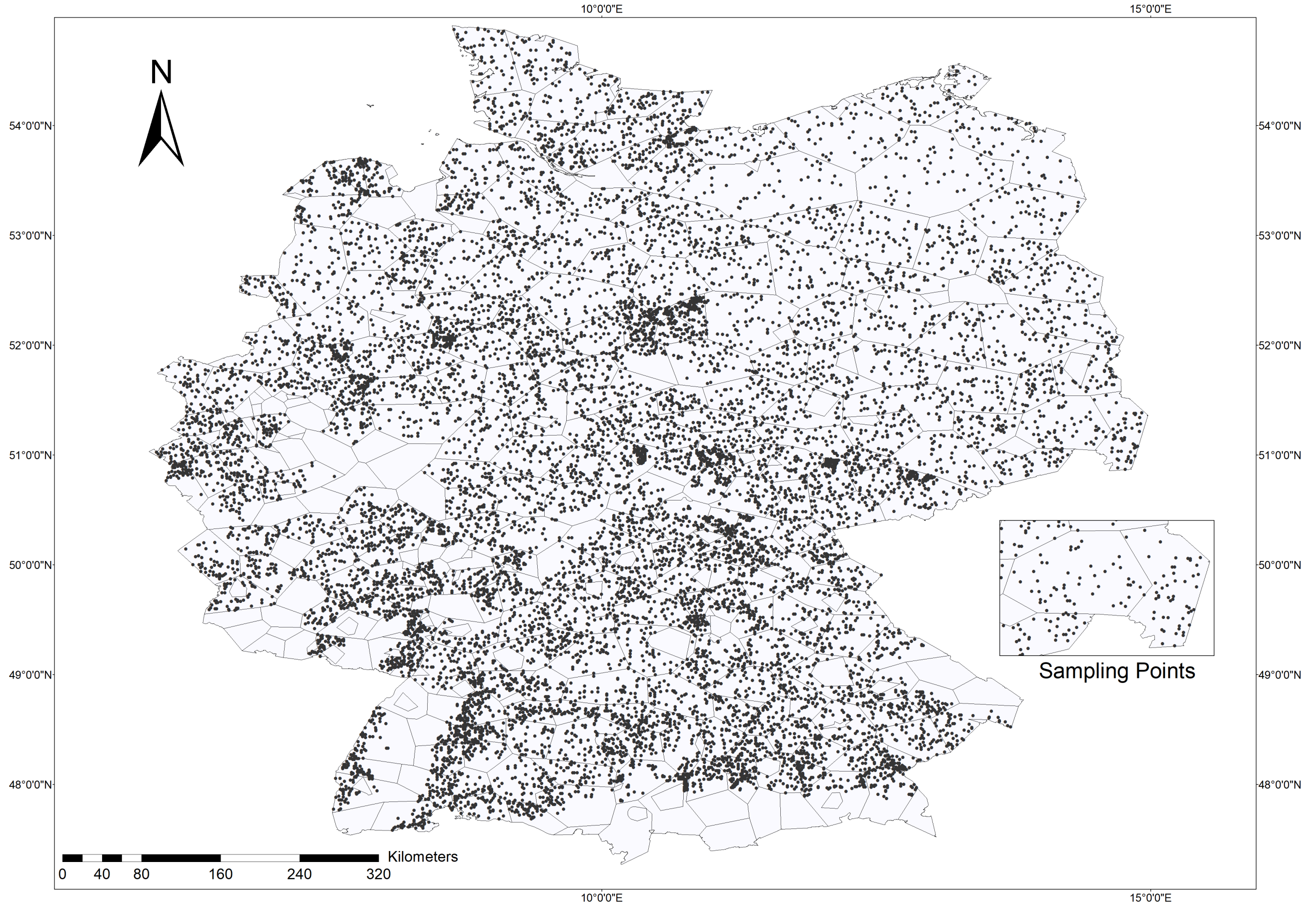

Figure S1. Sampling points for the weather and soil data collection in 271 counties across Germany. Figure was created using QGIS software version -3.18.3, https://qgis.org/en/site/).

Table S2. Summary statistics of winter wheat yield. The unit of yield is tons ha^{-1}.

Summary Statistics	Winter Wheat Yield
Total number of locations	271
Year range	1999-2019
Mean Yield	6.3
Standard deviation of yield	0.98
Minimum yield	2.2
Maximum yield	9.5
Number of weather components	6
Number of soil components	7
Number of phenology components	3
Number of observations	5692

Table S3. Summary statistics of the independent variables in the study

Variable	count	mean	std	min	25%	50%	75%	max
Precipitation (mm)	5691	14.30	3.07	6.79	12.06	14.06	16.21	29.33
TempMin (°C)	5691	4.44	1.10	1.46	3.62	4.41	5.18	8.29
TempMax (°C)	5691	12.99	1.18	9.45	12.13	12.88	13.73	17.12
Radiation (KJ m-2)	5691	10630.14	684.02	9082.78	10118.25	10548.04	11096.27	13140.76
RelHumCalc (%)	5691	0.73	0.02	0.68	0.71	0.73	0.74	0.79
Windspeed (ms-1)	5691	2.56	0.10	2.35	2.51	2.58	2.61	2.72
FloweringDOY (day)	5691	162.37	8.83	148.00	157.00	157.00	174.86	176.00
HarvestDOY (day)	5691	219.55	3.51	216.00	217.00	217.00	224.52	225.00
SowingDOY (day)	5691	278.56	5.43	271.00	271.60	281.00	281.00	293.00
LL (cm3cm-3)	5691	0.14	0.02	0.10	0.14	0.15	0.15	0.28
DUL (cm3cm-3)	5691	0.27	0.03	0.16	0.26	0.28	0.28	0.42
SAT (cm3cm-3)	5691	0.43	0.02	0.38	0.42	0.43	0.43	0.53
BD (gcm-3)	5691	2.18	0.64	0.49	1.73	2.16	2.50	4.39

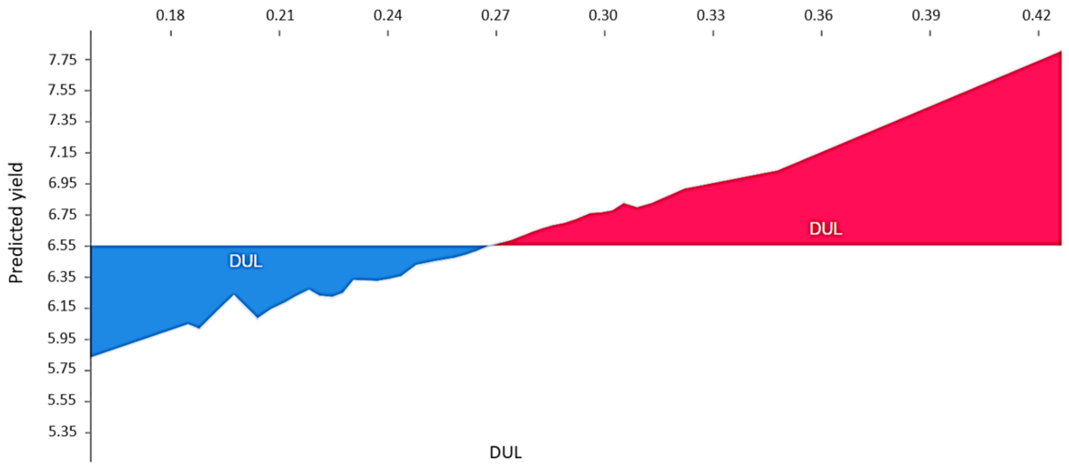

Figure S2. Force plot for crop available water at the field capacity (DUL) and predicted yield (in tons ha^{-1}) in 2019

Table S4. Hyperparameter settings of the baseline machine learning models used for predicting the winter wheat yield

Model	Parameters	Best Parameter
SVR	C	1
	Gamma	0.01
	Kernel type	Rbf
	Epsilon	0.15
KNN-regression	Leaf size	45
	Number of neighbors	3
	P	1
Lasso regression	alpha	0.0001
Ridge regression	alpha	0.01
	Solver	Auto
Regression tree	Criterion	Mse
	Maximum depth	8
Random Forest	Number of estimators	644
	Max. feature numbers	Sqrt
	Max. depth	57
	Min. samples split	5
	Min. samples leaf	1
	Bootstrap	FALSE
XGBoost	Max. depth	11
	Objective	[reg:squared error]
	regularization alpha	0.0001
	Min. child weight	5
	Gamma	0.05
	Learning rate	0.09
	Booster	Gbtree
	Subsample	0.6
	Column sample by tree	0.9
DNN	Number of layers	5
	Number of neurons	50
	Optimizer	Adam
	Batch size	8
	Number of epochs	50
	Activation function	ReLu
	Learning rate	0.0003

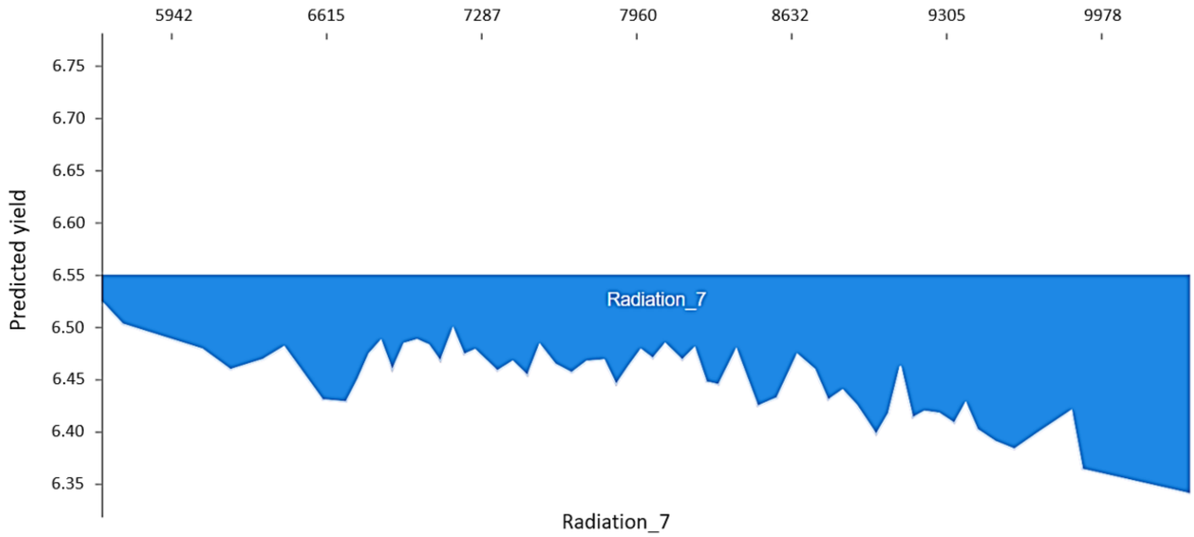

Figure S3. Force plot for radiation at week 7 (*Radiation*_7) and predicted yield (in tons ha^{-1}) in 2019

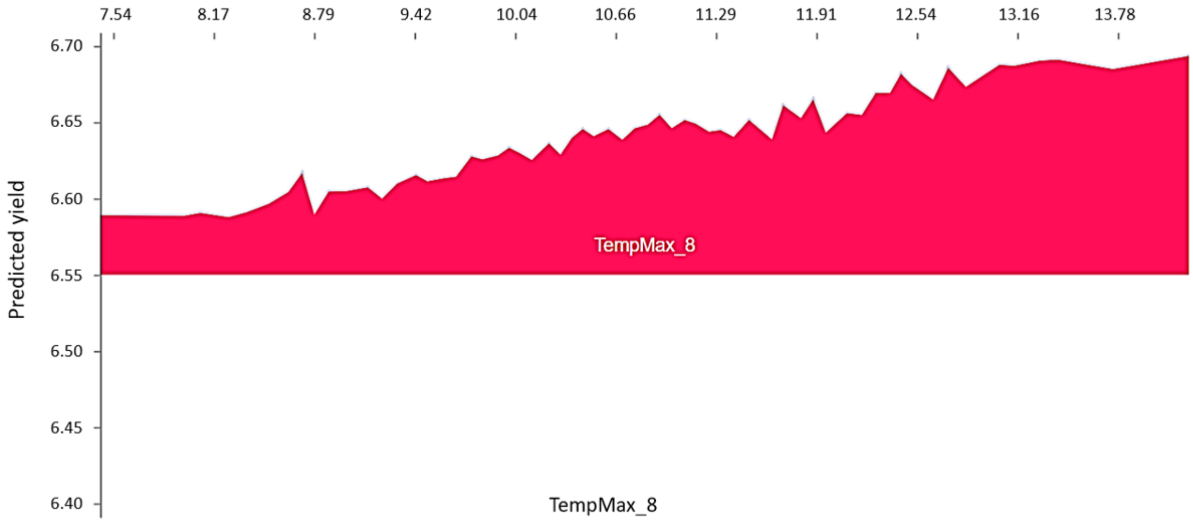

Figure S4. Force plot for the maximum temperature at week 8 (*TempMax*_8) and predicted yield (in tons ha^{-1}) in 2019

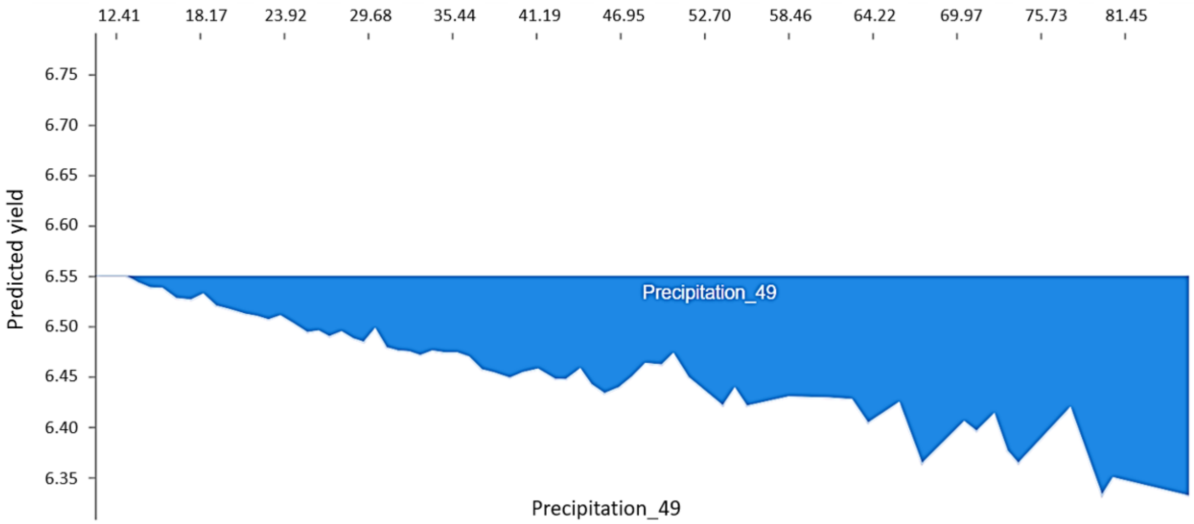

Figure S5. Force plot for precipitation at week 49 (*Precipitation*_49) and predicted yield (in tons ha^{-1}) in 2019

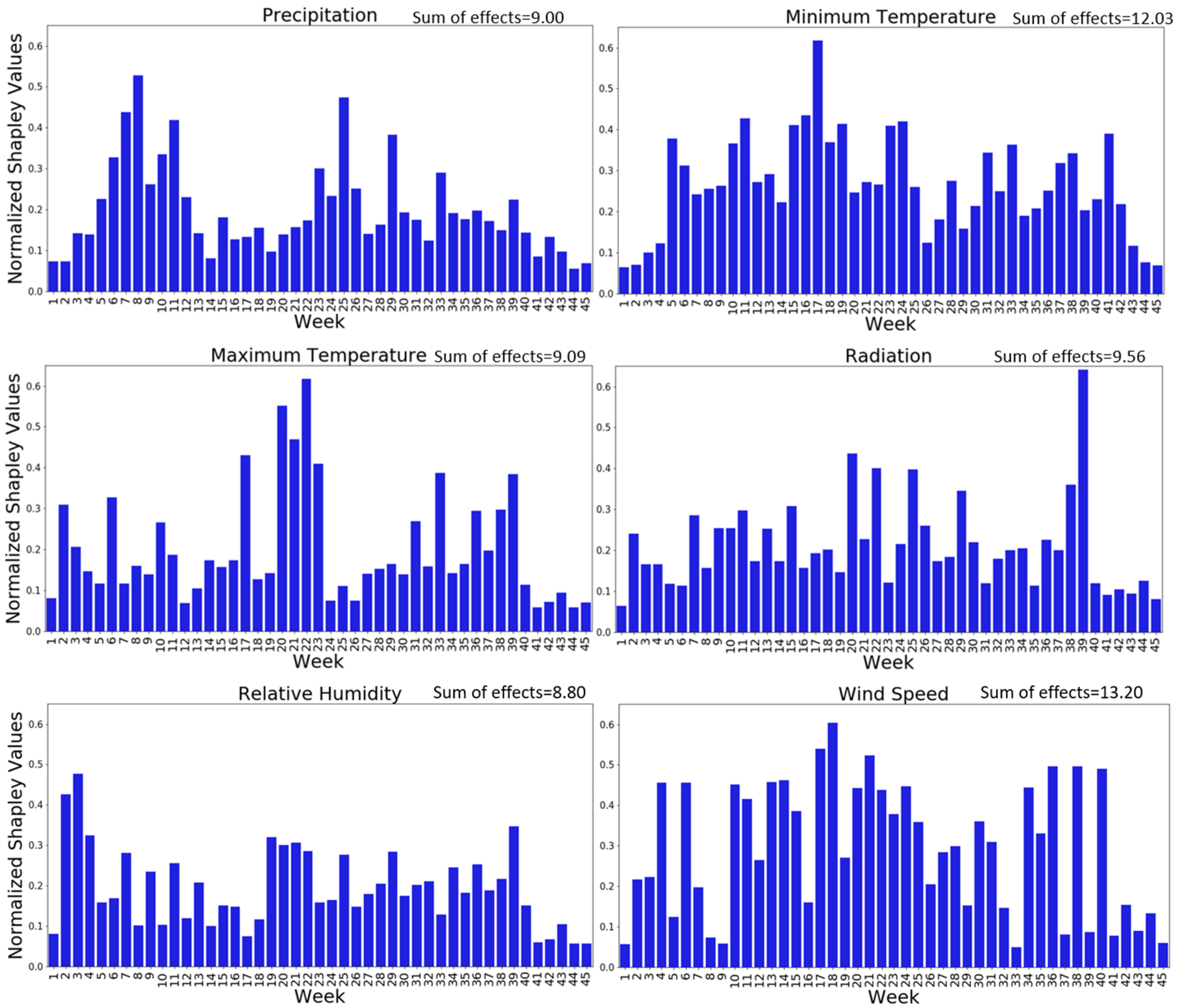

Figure S6. Estimated effects of six weather components on winter wheat measured for 45 weeks in 2019, starting from late Sept (week 1) and ending in mid-August (week 45). The vertical axes were normalized across all-weather components to make the effects comparable.
